# DNA barcode-based delineation of putative species: efficient start for taxonomic workflows

**DOI:** 10.1111/1755-0998.12233

**Published:** 2014-03-10

**Authors:** Mari Kekkonen, Paul D N Hebert

**Affiliations:** 1 Zoology Unit, Finnish Museum of Natural History, University of HelsinkiP.O. Box 17, FI-00014, Helsinki, Finland; 2 Biodiversity Institute of Ontario, University of GuelphGuelph, ON, N1G 2W1, Canada

**Keywords:** Australia, Automatic Barcode Gap Discovery, Barcode Index Number, DNA barcoding, General Mixed Yule-coalescent, Hypertrophinae

## Abstract

The analysis of DNA barcode sequences with varying techniques for cluster recognition provides an efficient approach for recognizing putative species (operational taxonomic units, OTUs). This approach accelerates and improves taxonomic workflows by exposing cryptic species and decreasing the risk of synonymy. This study tested the congruence of OTUs resulting from the application of three analytical methods (ABGD, BIN, GMYC) to sequence data for Australian hypertrophine moths. OTUs supported by all three approaches were viewed as robust, but 20% of the OTUs were only recognized by one or two of the methods. These OTUs were examined for three criteria to clarify their status. Monophyly and diagnostic nucleotides were both uninformative, but information on ranges was useful as sympatric sister OTUs were viewed as distinct, while allopatric OTUs were merged. This approach revealed 124 OTUs of Hypertrophinae, a more than twofold increase from the currently recognized 51 species. Because this analytical protocol is both fast and repeatable, it provides a valuable tool for establishing a basic understanding of species boundaries that can be validated with subsequent studies.

## Introduction

Species delimitation studies have traditionally focused on fine-tuning problematic complexes by compiling varied types of data (e.g. DNA sequences, morphological characters, karyotypes) and examining multiple individuals of each species. Although analyses of this type are appropriate for well-studied groups such as European butterflies (e.g. Dinca *et al*. 2011), baseline knowledge is much less for many taxonomic assemblages (Common 1990; Raven & Yeates 2007). As a result, there is a critical need for an approach which enables the simultaneous analysis of large numbers of putative species, even if it delivers a less precise outcome.

Prior work has shown that preliminary species delineation can often be achieved by analysing single-locus data from suitable genomic regions, such as the 648bp region of the mitochondrial cytochrome *c* oxidase subunit I selected as DNA barcodes (Hebert *et al*. 2003a,b; Hausmann *et al*. 2011; Collins *et al*. 2012a; Magnacca & Brown 2012). Because the use of any mtDNA marker risks exposure to complications such as introgression and incomplete lineage sorting, particularly for closely related species (Funk & Omland 2003; Dupuis *et al*. 2012; Talavera *et al*. 2013), sequence clusters revealed by the analysis of single-locus data should be considered as operational taxonomic units (OTUs). DNA barcode-based delimitation of species is best viewed as a quick start for the taxonomic process.

Several analytical methods support species delineation with single-locus data, partitioning sequences into genetic clusters without adopting a rigid sequence threshold. One popular approach, the General Mixed Yule-coalescent (GMYC; Pons *et al*. 2006; Fujisawa & Barraclough 2013), takes advantage of both Yule’s (1924) and Kingman’s (1982) models for calculating the maximum-likelihood solution for the transition point between the speciation and coalescence processes on an ultrametric gene tree. Under GMYC, the number of OTUs (putative species) equals the number of lineages crossing the threshold line. Although Monaghan *et al*. (2009) modified the original single-threshold model to incorporate variable threshold values throughout a tree, the single-threshold approach is generally preferred (e.g. Brewer *et al*. 2012; Paz & Crawford 2012).

Two methods designed for the analysis of DNA barcode data, Automatic Barcode Gap Discovery (ABGD; Puillandre *et al*. 2012a) and Barcode Index Number System (BIN; Ratnasingham & Hebert 2013) employ a different approach. Both ABGD and BIN apply clustering algorithms to distinguish partitions in the genetic distances among a group of individuals, using a two-phased procedure to create a final array of OTUs. ABGD first divides the data into groups based on a statistically inferred barcode gap and then recursively applies the same procedure to the groups obtained in the first step. By comparison, the BIN approach initially employs single linkage clustering coupled with a 2.2% threshold to establish preliminary OTU boundaries followed by secondary analysis using Markov clustering. The biphasic process has the same goal for both methods: improving and, if needed, redefining groups recovered in the first phase.

The congruence among the three methods can be viewed as supporting the robustness of any particular OTU due to their differing analytical approaches and theoretical basis (Carstens *et al*. 2013). Furthermore, comparison of these methods aids understanding of their tendency to either split or merge clusters. Their performance was contrasted in an earlier study that examined eight data sets covering several taxonomic groups including three well-studied lepidopteran assemblages (Ratnasingham & Hebert 2013). This analysis indicated that the three approaches had similar success in recognizing OTUs that matched known species, but that none delivered perfect correspondence. The results from GMYC and ABGD have been compared in several other studies with general congruence although GMYC tends to deliver a higher OTU count than ABGD, especially as the number of species rises (Jörger *et al*. 2012; Pantaleoni & Badano 2012; Paz & Crawford 2012; Puillandre *et al*. 2012b; Tang *et al*. 2012; Hendrixson *et al*. 2013; Weigand *et al*. 2013). When these methods have been examined for their capacity to re-cover previously recognized species, the results have been divergent with preference towards GMYC in some cases (Tang *et al*. 2012) and ABGD in others (Paz & Crawford 2012).

If congruence is viewed as a measure of the robustness of any OTU, how should cases of discordance be interpreted? Conservative (Weigand *et al*. 2013) and minimum consensus (Jörger *et al*. 2012) approaches have been adopted in the past, but both discard much information. Because the proportion of abandoned data will likely increase as the number of species rises (because there will be more chances for mismatches), such approaches are not ideal for large data sets. In this study, we employ three criteria derived from different species concepts to aid a final decision on the status of any ‘controversial’ OTU: monophyly, diagnostic characters (nucleotide substitutions) and the sympatry of sister OTUs. The inclusion of these parameters reflects their importance as a criterion for one or more species concepts. For example, the phylogenetic species concept (Rosen 1979; Mishler & Donoghue 1982; Donoghue 1985; Mishler 1985) requires that members of a species form a monophyletic unit, motivating our inclusion of this criterion. Another variant of the phylogenetic species concept demands that each species possess diagnostic characters lacking from its sister taxa (Nelson & Platnick 1981; Cracraft 1983; Nixon & Wheeler 1990), justifying our test for such characters. Finally, the biological species concept (Dobzhansky 1937; Mayr 1940; Wright 1940) requires that members of a species comprise a reproductively isolated group, a criterion that can only be tested in nature when species are sympatric (Coyne & Orr 2004). Despite their varied perspectives, different species concepts usually deliver congruent decisions when the taxa being considered have evolved independently for a substantial interval (de Queiroz 2005).

**Figure 1:**
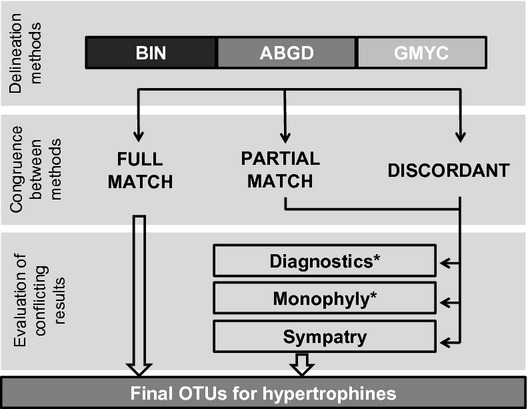
A flowchart describing the protocol, starting with the use of three delineation methods and followed by the division of resultant OTUs into three categories. OTUs assigned to FULL MATCH are included within the final OTU counts, while OTUs in the PARTIAL MATCH and DISCORDANT categories are evaluated against three criteria: sympatry, diagnostic characters, and monophyly (the latter two were tested, but uninformative). Description of FULL MATCH, PARTIAL MATCH, and DISCORDANT categories are provided in the Material and Methods.

This study represents one of the first efforts to use DNA barcode data as a taxonomic exploration tool, grouping specimens into OTUs that can be viewed as the first step towards a framework for subsequent phylogenetic and taxonomic work. The study focuses on the Hypertrophinae, a group of poorly known moths endemic to Australia. We employ a novel combination of methods to reach this goal, examining the congruence of OTUs resulting from three delimitation methods (GMYC, ABGD, BIN). We subsequently evaluate cases of discordance in OTU boundaries employing monophyly, diagnostic characters and sympatry as criteria for clarifying their status (Fig.1).

## Materials and methods

### Sampling

The Hypertrophinae was chosen for study due to its high endemism and many undescribed species (Common 1996). With the exception of two species from New Guinea, the group is only known from 51 described species in 12 genera endemic to Australia (Common 1980). The biology and distributions of its component species are very poorly known, and compilation of this information will be constrained until the taxonomy of the group is improved.

A total of 864 specimens of Hypertrophinae were analysed, covering all described Australian species, selecting representatives from across the known distribution of each taxon including all biogeographical regions of Australia (Ebach *et al*. 2013). A large proportion of these specimens were sampled in the Australian National Insect Collection (ANIC) during 2010 and 2011. Additional specimens were analysed from the Agricultural Scientific Collections Unit (ASCU); the Australian Museum, Sydney (AMS); the Biodiversity Institute of Ontario, University of Guelph (BIOUG); the Finnish Museum of Natural History, University of Helsinki (MZH), and the private collections of Graeme Cocks and Doug Hilton. Identifications follow original species descriptions (listed in Appendix S1, Supporting information) and taxonomic assignments for specimens in ANIC, mainly reflecting curatorial activity by Ian Common. No type specimens were examined.

### DNA extraction, PCR amplification and sequencing

DNA extraction, PCR and sequencing were performed at the Canadian Centre for DNA Barcoding following standard high-throughput protocols (deWaard *et al*. 2008). The first round of PCR employed the primers LepF1 and LepR1 (Hebert *et al*. 2003a) which generate a 658bp amplicon that spans the barcode region of CO1. In cases of failure, two additional PCR reactions were carried out to re-cover 306bp amplicon and 407bp amplicon using a standard primer set (Hajibabaei *et al*. 2006). If one of these reactions was successful, an effort was made to obtain a barcode compliant record (>497bp) by amplifying shorter regions of CO1 using the primer sets described in Hebert *et al*. (2013). All sequences were aligned using the BOLD Aligner in the Barcode of Life Data Systems (BOLD; Ratnasingham & Hebert 2007) and then inspected visually for stop codons and frameshift mutations in MEGA 5 (Tamura *et al*. 2011).

### Data analyses

Sequences were automatically assigned to a BIN on the BOLD Workbench v3.6 (http://www.boldsystems.org; analyses performed on 9 May 2013 and repeated on 8 December 2013) where assignments are easily visualized using the Taxon ID Tree. ABGD analyses were performed at the web interface (http://wwwabi.snv.jussieu.fr/public/abgd/, web version ‘April 11 2013’, performed on 31 August 2012, repeated on 6 December 2013; source code for ABGD is provided in Appendix S2, Supporting information) using a default value of relative gap width (X = 1.5) and both available distance metrics [JC69 (Jukes & Cantor 1969), K2P (Kimura 1980)] together with p-distance. All assignments for intraspecific divergence (*P*) values between 0.001 and 0.100 were recorded, while other parameter values employed defaults. The General Mixed Yule-coalescent (GMYC) method requires a fully resolved ultrametric gene tree as input for the analysis. We constructed a Bayesian inference tree in BEAST (Drummond *et al*. 2006; Drummond & Rambaut 2007) employing a Yule pure birth model (Gernhard 2008) tree prior. XML file (Appendix S3, Supporting information) was made with BEAUti v1.7.1 interface with the following settings: GTR+G+I substitution model, empirical base frequencies, four gamma categories, all codon positions partitioned with unlinked base frequencies and substitution rates. An uncorrelated relaxed lognormal clock model was used with rate estimated from the data and ucldmean parameter with uniform prior to value 0 as a lower and 10 as an upper boundary. All other settings were left as defaults. The length of MCMC chain was 40 000 000 sampling every 4000. All BEAST runs were executed in Bioportal (Kumar *et al*. 2009), and the ESS values and trace files of runs were evaluated in Tracer v1.5.0. Two independent runs were merged using LogCombiner v1.7.1 with 20% burn-in. Maximum clade credibility trees with a 0.5 posterior probability limit, and node heights of target tree were constructed in TreeAnnotator v1.7.1. Both single- and multiple-threshold GMYC analyses were conducted in R (R Core Team. 2012) using the APE (Paradis *et al*. 2004) and SPLITS (Ezard *et al*. 2009) packages (for R code used for GMYC analyses, see Appendix S4, Supporting information). GMYC analyses were performed with haplotype data collapsed in ALTER (Glez-Peña *et al*. 2010, performed on 11 December 2012). Maximum-likelihood analysis was also performed with haplotype data to compare the results of Bayesian inference and maximum likelihood using RAxML BlackBox (Stamatakis *et al*. 2008, performed on 12 May 2013) with GTR+G model and default bootstrap settings.

### Comparison of resulting OTUs

The congruence of the three species delimitation methods was evaluated by comparing the composition of the clusters recognized by each method. To aid comparison, the OTUs were divided into three categories: FULL MATCH where all methods generated the same partition, PARTIAL MATCH where two of three methods generated similar results and DISCORDANT where all three led to a different result.

OTUs in the PARTIAL MATCH and DISCORDANT categories were analysed for diagnostic characters between sister OTUs based on application of the phylogenetic species concept using function nucDiag in the R package SPIDER (Brown *et al*. 2012). This function only considers pure diagnostic characters *sensu* Sarkar *et al*. (2008). Although a search for diagnostic characters was conducted for all clusters, its validity is questionable for clusters with few representatives. In addition, monophyly over a NJ tree was studied with the function monophyly in SPIDER. To evaluate putative species status from the context of the biological species concept, we compared the range for members of each distinct OTU based on the coordinates for these specimens in BOLD. Sister OTUs were considered as sympatric when they occupied the same biogeographical region (terrestrial zoogeographical subregions in Ebach *et al*. 2013). Both range comparison and the search for diagnostic characters were conducted for pairs of sister taxa based on the topology of the Bayesian inference tree. For range estimation, all barcode compliant sequences of hypertrophines in BOLD were included for OTUs in the PARTIAL MATCH and DISCORDANT categories.

## Results

**Figure 2:**
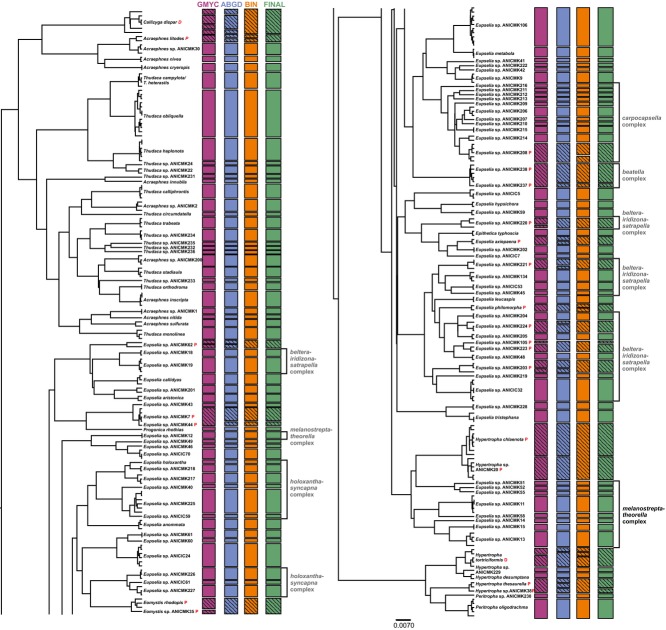
Bayesian inference gene tree with delineated OTUs. OTUs in the PARTIAL MATCH and DISCORDANT categories are marked with red letters P and D, respectively.

Sequence data were recovered from 702 of the 864 specimens, but some records from older specimens were incomplete. The collection year of successfully sampled specimens varied from 1958 to 2012, but most specimens were collected in the last decade (Fig. S5, Supporting information). Subsequent analysis of OTU diversity focused on 502 full-length (654bp as the BOLD aligner reduces the original length of 658bp by omitting the first and three last bases) barcode sequences which included 294 haplotypes. These records provided coverage for 47 of the 51 known hypertrophine species from Australia (*Oxytropha ametalla*, *Thudaca cymatistis*, *T. monolechria* and *T. ophiosema* lacked coverage). The sequences used here are publicly available on BOLD and GenBank (see Table S6, Supporting information for Accession nos; DOI: dx.doi.org/10.5883/DS-HOTUS). We only used full-length sequences to remove complications introduced by missing data. Overall pairwise distances (K2P) indicated a clear barcode gap between 0.01 and 0.05 (*n* = 65 536, mean = 0.104) (Fig. S7, Supporting information). A comparison between Bayesian inference (Fig.2) and maximum-likelihood (Fig. S8, Supporting information) gene trees did not reveal obvious differences in the OTUs.

**Figure 3:**
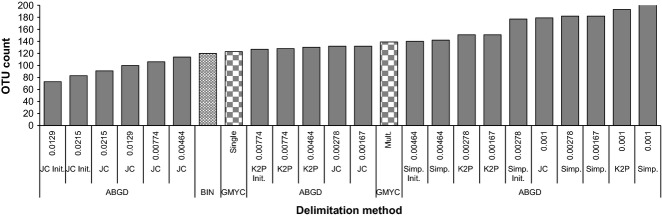
OTU counts resulting from three delineation methods. Figures below the results for ABGD indicate prior intraspecific divergence (*P*) values. The two OTU counts for GMYC result from single- and multiple-threshold models.

The count of OTUs varied from 73 to 222 with both the lowest and highest result produced by ABGD (Fig.3). ABGD analysis with JC69 produced two initial partitions with OTU counts of 73 (*P* = 0.0129) and 83 (*P* = 0.0215), whereas use of K2P returned only one initial value of 127 OTUs (*P* = 0.00774) (Table1). Because the use of p-distance produced strongly discordant outcomes with the initial partition including 140 OTUs (*P* = 0.00464) and 177 (*P* = 0.00278) OTUs (Table1), it was omitted. BIN (120 OTUs) and GMYC (123 OTUs) with a single-threshold model produced very similar results, and values close to the 127 OTUs obtained with ABGD and the initial partition of K2P. Similar to many earlier studies, the implementation of GMYC with a multiple-threshold model produced a higher OTU count (139) than the single-threshold model, but it failed to improve the fit of the GMYC model to the data (*χ*^2^ = 12.73, d.f. = 15, *P* < 0.62) (Table2). Also, the likelihood-ratio test rejected the null model denoting the presence of more than one species in the data (Table2).

**Table 1:** Results of the Automatic Barcode Gap Discovery (ABGD) analyses

Subst. model	X	Partition	Prior intraspecific divergence (*P*)							
			0.0359	0.0215	0.0129	0.00774	0.00464	0.00278	0.00167	0.001
Simple	1.5	Initial				0	140	177	177	177
		Recursive				0	142	182	182	222
JC	1.5	Initial	0	83	73	73	73	73	73	73
		Recursive	0	91	100	106	114	132	132	179
K2P	1.5	Initial			0	127	127	127	127	127
		Recursive			0	128	130	151	151	193

X, relative gap width; Simple, p-distance; JC69, Jukes-Cantor substitution model; K2P, Kimura 2-parameter substitution model.

**Table 2:** Results of the General Mixed Yule-coalescent (GMYC) analyses

Analysis	Clusters (CI)	Entities (CI)	Likelihood_null_	Likelihood_GMYC_	Likelihood ratio	Threshold
Single	76 (75–77)	123 (120–130)	2495.37	2539.71	88.68^*^^*^^*^	−0.003837296
Multiple	70 (70–72)	139 (139–145)	2495.37	2541.51	92.28^*^^*^^*^	−0.0101113
						−0.003837296
−0.003271336
−0.002950595
−0.001794289

Clusters, OTUs delineated by GMYC with more than one specimen; Entities, singleton OTUs delineated by GMYC; CI, confidence interval; Likelihood_null_, likelihood of the null model; Likelihood_GMYC_, likelihood of the GMYC model; Threshold, the threshold between speciation and coalescence processes; Single, single-threshold model; Multiple, multiple-threshold model; ^*^^*^^*^P < 0.001.

To examine the congruence of putative species, we assigned each cluster into one of three categories (FULL MATCH, PARTIAL MATCH, DISCORDANT). In making these assignments, we only considered results from the initial partition with K2P from ABGD as the OTU count was closest to those from the other methods. The results obtained with JC69 were excluded due to the extensive merging of clusters which was in strong conflict with the results from the other two methods. From the two GMYC analyses, we only included the single threshold for the above-mentioned reason. Comparison of the assignments showed that 96 OTUs (80%) were recognized by all three methods (i.e. FULL MATCH). Another 22 OTUs (18.3%) were PARTIAL MATCHES, while only two OTUs (1.7%) were DISCORDANT (splits within *Hypertropha tortriciformis* and *Callizyga dispar*) (Fig.2).

Diagnostic characters were discovered for all different OTU boundaries within the PARTIAL MATCH and DISCORDANT categories, although this outcome was undoubtedly due, at least in part, to the fact that most conflicting OTUs were represented by few individuals. The test for monophyly revealed that the OTUs delimited by ABGD and BIN each included one paraphyletic group (*Eupselia* sp. ANICMK238 of *beatella* complex*),* whereas two groups were paraphyletic with GMYC (*Eupselia* sp. ANICMK105 of *beltera*-*satrapella-iridizona* complex and a split from *Hypertropha tortriciformis*).

**Figure 4:**
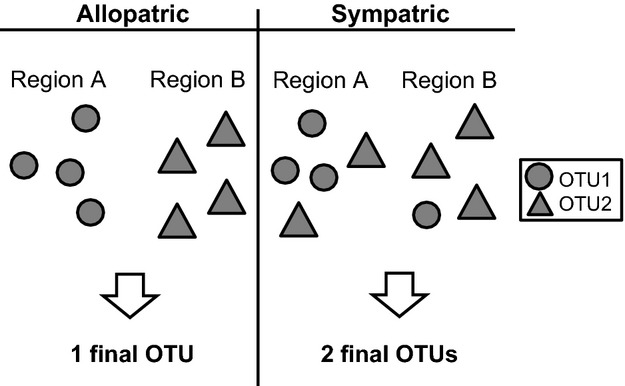
Sympatry criterion for sister OTUs in PARTIAL MATCH category. OTUs found in separate biogeographical regions are merged to form one final OTU while sister OTUs sympatric in one or several regions are recognized as two final OTUs.

The two OTUs (*Hypertropha tortriciformis* and *Callizyga dispar*) in the DISCORDANT category may well include more than one species, but each was treated as a single OTU due to the conflicting results. The status of the 22 OTUs in the PARTIAL MATCH category was evaluated by examining the sympatry criterion for sister groups (Fig.4) similar to the integrative taxonomic approach (ITAX) of Miralles & Vences (2013). Eight of the OTUs partitioned by one of the methods failed to meet the sympatry criterion. Three of these eight OTUs included allopatric subgroups (i.e. restricted to different biogeographical regions), while five other OTUs were represented by a single specimen allopatric from a sister OTU composed of multiple specimens. All eight of these PARTIAL MATCHES were treated as a single OTU on the conservative presumption that the sequence divergence apparent between their allopatric lineages reflected phylogeographic variation in a single taxon. The remaining 14 PARTIAL MATCHES involved cases of sister OTUs which occurred in sympatry, so they were recognized as distinct (*Eomystis rhodopis* and *Eomystis* sp. ANICMK35; *Eupselia* sp. ANICMK237 and *E*. sp. ANICMK238 of *beatella* complex; *E.* sp. ANICMK208 and *E.* sp. ANICMK104 of *carpocapsella* complex; *E.* sp. ANICMK105 and *E.* sp. ANICMK223 of *E. satrapella-E. beltera-E. iridizona*; *E.* sp. ANICMK44 and *E.* sp. ANICMK7; *Hypertropha chlaenota* and *H*. sp. ANICMK20; *H. thesaurella* and *H*. sp. ANICMK38).

A final count of 120 putative species was obtained by recognizing FULL MATCH clusters (96) as distinct OTUs, and augmenting this total with those from the PARTIAL MATCH (3 + 5 allopatric, 14 sympatric) and DISCORDANT (2) categories.

## Discussion

### Estimating the number of Australian species of Hypertrophinae

The 502 specimens of Hypertrophinae examined in this study include 120 OTUs that are likely to represent distinct species. Because four known, morphologically distinctive species were not included in our study, the probable species count is at least 124, a more than two-fold increase from the currently recognized fauna. Because nearly 50% of the OTUs in this study were represented by just one or two individuals, it is likely that many additional taxa await discovery. An accumulation curve for OTUs (Fig. S9, Supporting information) shows reduced steepness, but indicates the likely presence of additional species. However, based on current results, it is already clear that two closely related genera, *Allotropha* and *Eupselia*, will rise in diversity (19 current species vs. 78 OTUs). While *Eupselia carpocapsella* provides a particularly striking example of cryptic species with 12 OTUs, four other lineages (*Allotropha percussana*-*Eupselia aristonica*, *E. holoxantha-E. syncapna, E. satrapella-E. beltera-E. iridizona* and *E. melanostrepta-E. theorella*) also likely form multispecies complexes. Interestingly, many of these complexes show polyphyly in the Bayesian gene tree (Fig.2), a result which might be an artefact of the gene tree, but the situation certainly calls for further investigation. Two other genera also appear to include unrecognized species with *Acraephnes* rising from 7 to 11 OTUs and *Thudaca* from 15 to 20 OTUs (*Thudaca crypsidesma* and *T. mimodora* were probably analysed, but none of the OTUs was assigned to these species because they lack clear morphological diagnostics). *Thudaca heterastis* showed no sequence difference from *T. campylota,* so these taxa may be synonyms and were treated as one OTU. OTUs were also added to *Hypertropha* (from 4 to 7), *Peritropha* (from 1 to 2) and *Eomystis* (from 1 to 2). No evidence of unrecognized species was obtained in the other four genera (*Callizyga*, *Epithetica*, *Oxytropha* and *Progonica*).

### Comparing the performance of OTU delineation methods

ABGD, GMYC and BIN showed good concordance with the same assignment for 80% of OTUs, supporting earlier studies (Puillandre *et al*. 2012b; Ratnasingham & Hebert 2013). However, congruence would have been much lower if other outcomes of ABGD were included. For example, the initial partition with JC69 merged many clusters, while recursive partitions with JC69 and K2P created many splits. This difference between distance metrics is strongly discordant from the results obtained by Collins *et al*. (2012b), a situation requiring further investigation. We adopted the initial partition of ABGD with K2P due to its closer correspondence with the results from BIN and GMYC, simplifying the comparison. Although recursive partitions of ABGD were excluded from the correspondence check, they revealed subgroups which may be useful in certain taxonomic contexts.

Automatic Barcode Gap Discovery generates diverse outcomes, and it is difficult to select the most appropriate one. Puillandre *et al*. (2012a) proposed adoption of a single value of *P* = 0.01 as it produced the strongest congruence with previous studies examining the same data with different approaches. In our analysis, this value was only produced by JC69, but the OTUs with this distance metric showed strong discordance to those obtained with the other methods. Because we selected the outcome from ABGD which delivered the closest OTU count to the other two methods, our test for the robustness of OTU boundaries (i.e. all methods assigning particular specimens to the same OTU) is partially compromised. However, it needs emphasis that the overall OTU count and the specimens composing each OTU are not strictly associated. As results from the three approaches diverged in 20% of all OTUs, they certainly provide some insights into the stability of the OTUs. However, more investigations are needed to strengthen the use of ABGD so that the adoption of a particular value of *P* is made without the *a posteriori* approach employed in this study.

General Mixed Yule-coalescent has a strong theoretical basis, but it typically generates more OTUs than other methods (Esselstyn *et al*. 2012; Paz & Crawford 2012; Sauer & Hausdorf 2012; Miralles & Vences 2013; Talavera *et al*. 2013) and errors in the ultrametric gene tree that underpins the analysis will influence final results. In addition, GMYC calculations are very time-consuming for large data sets due to their requirement for an input tree (the multiple-threshold model is a particular challenge). BIN is the fastest and most user-friendly of the methods as it delivers only one result, making clear the OTU boundaries which need evaluation. All three methods have the tendency to split outliers, but, as indicated above, we treated these as a probable artefact of geographical distance and not as reflective of a species boundary.

Employing three analytical approaches improves confidence in the validity of OTUs delineated by all approaches, although Carstens *et al*. (2013) encourage using even more methods. Conflicting results can be viewed as indicators for OTU boundaries which deserve detailed inspection. The use of several methods does have one disadvantage; it increases the complexity and time required for OTU evaluation.

Adequate sample sizes are critical for any effort to delineate species (e.g. Lohse 2009). If the current species count (51) for Australian Hypertrophinae was complete, our analysis of 502 specimens would have provided nearly 10× coverage if each taxon had equal representation. However, 46% of the OTUs (55 of 120) in our analyses were represented by just one or two specimens, reflecting the commonness of rarity in nature (Lim *et al*. 2012). This fact emphasizes the need for analytical methods that deal effectively with low taxon coverage. Because simulation studies indicate that ABGD performs poorly unless there are 3–5 samples per species (Puillandre *et al*. 2012a), its use for explorations of species diversity in poorly known groups is problematic, because the number of samples per species is impossible to know *a priori*. Interestingly, despite this limitation, one analytical option of ABGD generated results that were relatively congruent with other methods despite the low numbers of specimens.

### Criteria for discordant OTU boundaries

To be useful, each test criterion requires differences between OTUs assigned to the PARTIAL MATCH and DISCORDANT categories. For example, cases of monophyly or the presence of diagnostic characters would support the validity on a controversial OTU, while the detection of paraphyly or the lack of diagnostic characters would not. Because all discordant OTUs in our study possessed diagnostic nucleotide substitutions, this criterion did not help to clarify their status. This criterion may be useful in other situations, but its utility will often be compromised by the rarity of many taxa. The test for monophyly revealed few cases of paraphyly, so it was also of little value in clarifying OTU boundaries. Apparently, the three delimitation methods typically recognize breaks in sequence space associated with monophyly, so secondary inspection reveals few exceptions.

By contrast, the sympatry criterion provided a useful tool for the evaluation of conflicts in OTU boundaries. When two allopatric populations are only assigned to distinct OTUs by certain methods, their status as distinct species becomes questionable (e.g. Mutanen *et al*. 2012). By comparison, when sister OTUs show range overlap, this provides presumptive evidence for their reproductive isolation although it should be confirmed by nuclear markers. We add two provisos. Because sympatry was imprecisely evaluated in this study, sister OTUs viewed as sympatric may actually be microallopatric. Conversely, because sampling efforts were not comprehensive, future sampling may reveal that sister OTUs currently viewed as allopatric are actually sympatric.

### Delineating species with DNA barcodes

This study describes an efficient protocol for obtaining an initial taxonomic framework. Puillandre *et al*. (2012b) adopted a more complex strategy for delimiting species of marine molluscs which coupled testing initial OTUs (primary species hypotheses) for differences in morphology, sequence divergence at additional loci and the dispersal capacity of larvae before creating secondary species hypotheses. Riedel *et al*. (2013) presented an even more complex approach covering the whole taxonomic procedure. Our scheme has the advantage of keeping the initial step of OTU designation separate from the detailed analysis required for full-blown taxonomic characterization. Because the varying steps in species delineation require different sampling strategies and types of data, the primary delineation of OTUs with single-locus data has the advantage of employing one extensive data set with clearly defined criteria to produce a stable outcome.

We emphasize that the delimitation of putative species based on DNA barcode data not only increases objectivity, but accelerates work on poorly studied groups and enables inexperienced taxonomists to make a valuable contribution. As many groups of arthropods lack expert taxonomists, the need to recruit new experts is obvious and barcode-based approaches provide an easy path for initial engagement. Even without detailed study, an accurate estimate of the species count is obtained through the simple algorithmic processing of barcode data. While decisions based on analysis of single-locus mtDNA data and on small sample sizes do pose interpretational risks, they are inconsequential if the outcome is viewed as a scaffold for taxonomy rather than as the sole criterion for species description.

## Notes

M.K. designed the study, sampled most of the specimens at ANIC, analysed the data, and wrote the manuscript. P.D.N.H. was responsible for the sequence analysis and wrote the manuscript.

## Data Accessibility

DNA sequences: GenBank accessions see Table S6, Supporting information.

BOLD data set ‘Hypertrophinae OTUs’ (DS-HOTUS) DOI: http://dx.doi.org/10.5883/DS-HOTUS.

## Supporting Information

Appendix S1 The reference list of original species descriptions.

Appendix S2 The source code for ABGD. Downloaded from http://wwwabi.snv.jussieu.fr/public/abgd/ on 6 December 2013.

Appendix S3 XML file for BEAST.

Appendix S4 R code used for GMYC analyses.

Fig S5 The collection years of specimens with barcode sequences (any length).

Table S6 Samples used for OTU delineation. OTU names, BOLD Sample and Process IDs, GenBank accessions and storing institutions are provided.

Fig S7 Pairwise distances (K2P) for 502 full-length (654bp) DNA barcode sequences from Australian Hypertrophinae.

Fig S8 Maximum-likelihood tree with bootstrap values.

Fig S9 An accumulation curve of putative species (OTUs).
