# Supplementary material for: DNA barcode-based delineation of putative species: efficient start for taxonomic workflows
Source: Mol Ecol Resour. 2014 Mar 10;14(4):706–15. doi: 10.1111/1755-0998.12233 (PMC4264940; doi:10.1111/1755-0998.12233)
Supplement: Appendix S1 — The reference list of original species descriptions. [file men0014-0706-SD1.docx]

Diakonoff A (1954) *Peritropha oligodrachma* spec. nov. *Proceedings of the Koninklijke Nederlandse Akademie van Wetenschappen*, **C57**, 280.

Guenée M (1852) 964. *Heliodes tortriciformis* Gn. In: *Hist. Nat. Insectes (Lépid.)* (Boisduval, Guenée), **6**, 198.

Lower OB (1894) *Eupselia holoxantha*, n. sp. *Transactions and Proceedings and Report of the Royal Society of South Australia*, **18**, 105.

Lower OB (1899) *Eupselia iridizona*, n. sp. *Proceedings of the Linnean Society of New South Wales*, **24**, 115.

Lower OB (1902) *Eupselia philomorpha*, n. sp. *Proceedings of the Linnean Society of New South Wales*, **26**, 670.

Meyrick E (1880) *Eups. aristonica* n. sp. *Proceedings of the Linnean Society of New South Wales*, **5**, 218.

Meyrick E (1880) *Eups. melanostrepta*, n. sp. *Proceedings of the Linnean Society of New South Wales*, **5**, 223.

Meyrick E (1880) *Eups. satrapella*, n. sp. *Proceedings of the Linnean Society of New South Wales*, **5**, 220.

Meyrick E (1880) *Eups. theorella*, n. sp. *Proceedings of the Linnean Society of New South Wales*, **5**, 222.

Meyrick E (1880) *Hyp. thesaurella*, n. sp. *Proceedings of the Linnean Society of New South Wales*, **5**, 209.

Meyrick E (1887) *Hyper. chlaenota*, n. sp. *Proceedings of the Linnean Society of New South Wales*, **1**, 1042.

Meyrick E (1888) 525. (26a.) *Eom. rhodopis*, n. sp. *Proceedings of the Linnean Society of New South Wales*, **2**, 932.

Meyrick E (1893) 142. *Thud. heterastis*, n. sp. *Proceedings of the Linnean Society of New South Wales*, **7**, 571.

Meyrick E (1893) 143. *Thud. campylota*, n. sp. *Proceedings of the Linnean Society of New South Wales*, **7**, 572.

Meyrick E (1893) 144. *Thud. crypsidesma*, n. sp. *Proceedings of the Linnean Society of New South Wales*, **7**, 572.

Meyrick E (1893) 145. *Thud. haplonota*, n. sp. *Proceedings of the Linnean Society of New South Wales*, **7**, 573.

Meyrick E (1893) 146. *Thudaca mimodora*, n. sp. *Proceedings of the Linnean Society of New South Wales*, **7**, 574.

Meyrick E (1893) 148. *Thud. calliphrontis*, n. sp. *Proceedings of the Linnean Society of New South Wales*, **7**, 576.

Meyrick E (1893) 149. *Thud. ophiosema*, n. sp. *Proceedings of the Linnean Society of New South Wales*, **7**, 576.

Meyrick E (1893) 150. *Thud. cymatistis*, n. sp. *Proceedings of the Linnean Society of New South Wales*, **7**, 577.

Meyrick E (1893) 151. *Thud. orthodroma*, n. sp. *Proceedings of the Linnean Society of New South Wales*, **7**, 577.

Meyrick E (1893) 152. *Thud. stadiaula*, n.sp. *Proceedings of the Linnean Society of New South Wales*, ***7***, 578.

Meyrick E (1893) 153. *Thud. trabeata*, n. sp. *Proceedings of the Linnean Society of New South Wales*, ***7*,** 578.

Meyrick E (1906) *Eupselia hypsichora*, n. sp. *Transactions and Proceedings and Report of the Royal Society of South Australia*, **30**, 39.

Meyrick E (1906) *Eupselia leucaspis*, n. sp. *Transactions and Proceedings and Report of the Royal Society of South Australia*, **30**, 38.

Meyrick E (1906) *Eupselia trithrona*, n. sp. *Transactions and Proceedings and Report of the Royal Society of South Australia*, **30**, 39.

Meyrick E (1906) *Hypertropha rhothias*, n. sp. *Transactions and Proceedings and Report of the Royal Society of South Australia*, **30**, 51.

Meyrick E (1906) *Hypertropha zophodesma*, n. sp. *Transactions and Proceedings and Report of the Royal Society of South Australia*, **30**, 51.

Meyrick E (1907) 127. *A. sulfurata*, n.sp. *Proceedings of the Linnean Society of New South Wales*, **32**, 84.

Meyrick E (1915) *Eupselia callidyas*, n. sp. *Exotic Microlepidoptera*, **1**, 306.

Meyrick E (1915) *Eupselia tristephana*, n. sp. *Exotic Microlepidoptera*, **1**, 306.

Meyrick E (1920) *Eupselia syncapna*, n. sp. *Exotic Microlepidoptera*, **2**, 317.

Turner AJ (1894) *Callizyga dispar*, n. sp. *Transactions and Proceedings and Report of the Royal Society of South Australia*, **18**, 132.

Turner AJ (1898) *Eupselia anommata*, n. sp. *Transactions and Proceedings and Report of the Royal Society of South Australia*, **22**, 204.

Turner AJ (1898) *Hypertropha ametalla*, n. sp. *Transactions and Proceedings and Report of the Royal Society of South Australia*, **22**, 202.

Turner AJ (1923) *Epithetica typhoscia*, n. sp. *Transactions and Proceedings and Report of the Royal Society of South Australia*, **47**, 165.

Turner AJ (1927) *Thudaca innubila*, n. sp. *Papers and proceedings of the Royal Society of Tasmania*, 154.

Turner AJ (1947) 2196. *Eupselia beltera*, n. sp. *Proceedings of the Linnean Society of New South Wales*, **72**, 149.

Turner AJ (1947) 2197. *Eupselia axiepaena*, n. sp. *Proceedings of the Linnean Society of New South Wales*, **72**, 149.

Turner AJ (1947) 2204. *Eupselia metabola*, n. sp. *Proceedings of the Linnean Society of New South Wales*, **72**, 149.

Turner AJ (1947) 2208. *Progonica niphostibes*, n. sp. *Proceedings of the Linnean Society of New South Wales*, **72**, 150.

Turner AJ (1947) 2226. *Thudaca monolechria*, n. sp. *Proceedings of the Linnean Society of New South Wales*, **72**, 152.

Turner AJ (1947) 2235. *Thudaca cryeropis*, n. sp. *Proceedings of the Linnean Society of New South Wales*, **72**, 153.

Turner AJ (1947) 2237. *Thudaca litodes*, n. sp. *Proceedings of the Linnean Society of New South Wales*, **72**, 153.

Turner AJ (1947) 2238. *Acraephnes nivea*, n. sp. *Proceedings of the Linnean Society of New South Wales*, **72**, 153.

Turner AJ (1947) 2239. *Acraephnes nitida*, n. sp. *Proceedings of the Linnean Society of New South Wales*, **72**, 153.

Turner AJ (1947) 2242. *Haereta inscripta*, n. sp. *Proceedings of the Linnean Society of New South Wales*, **72**, 154.

Walker F (1863) 3. *Orosana desumptana*. *List of the specimens of lepidopterous insects in the collection of the British Museum*, **28**, 460.

Walker F (1864) 1. *Thudaca obliquella*. *List of the specimens of lepidopterous insects in the collection of the British Museum*, **29**, 825.

Walker F (1864*) Orosana* ? *beatella*. *List of the specimens of lepidopterous insects in the collection of the British Museum*, **30**, 999.

Walker F (1864) *Orosana* ? *carpocapsella*. *List of the specimens of lepidopterous insects in the collection of the British Museum*, **30**, 998.

Walker F (1864) *Orosana* ? *percussana*. *List of the specimens of lepidopterous insects in the collection of the British Museum*, **30**, 998.

Walker F (1864) *Tonza* ? *circumdatella*. *List of the specimens of lepidopterous insects in the collection of the British Museum*, **30**, 1012.

Walker F (1865) *Anthoecia divitiosa*. *List of the specimens of lepidopterous insects in the collection of the British Museum*, **33**, 771.
