# Supplementary figures and images for: DNA barcode-based delineation of putative species: efficient start for taxonomic workflows
Source: Mol Ecol Resour. 2014 Mar 10;14(4):706–15. doi: 10.1111/1755-0998.12233 (PMC4264940; doi:10.1111/1755-0998.12233)

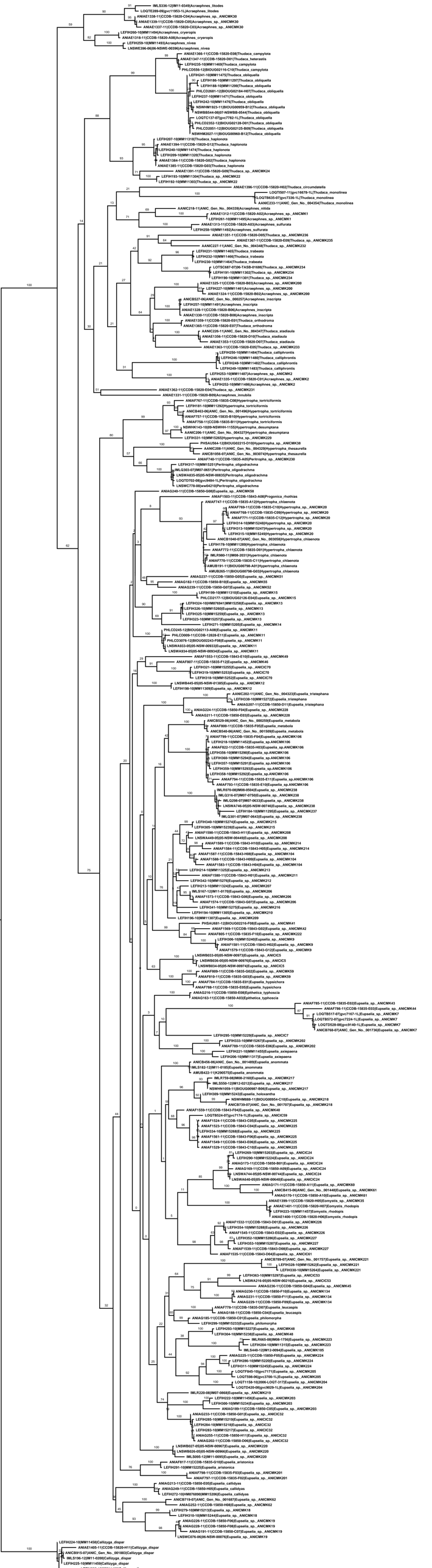

Supplement: Appendix S1 — The reference list of original species descriptions. [file men0014-0706-SD1.pdf]

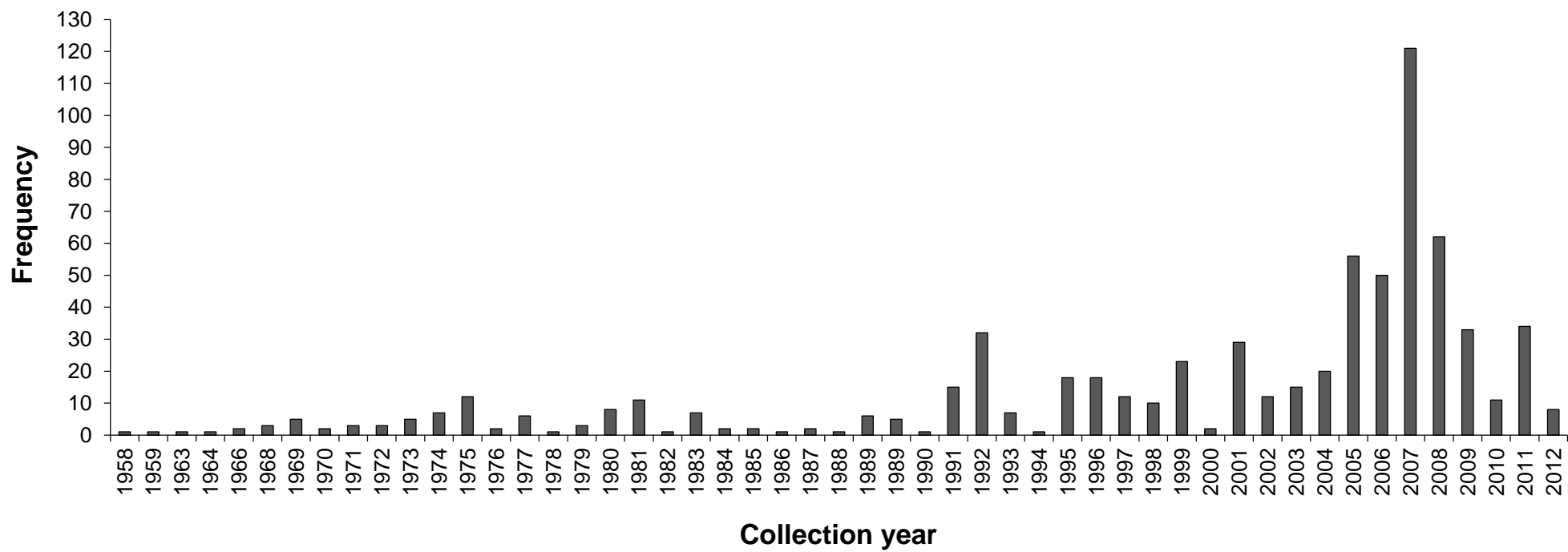

Supplement: Fig S5 — The collection years of specimens with barcode sequences (any length). [file men0014-0706-SD5.pdf]

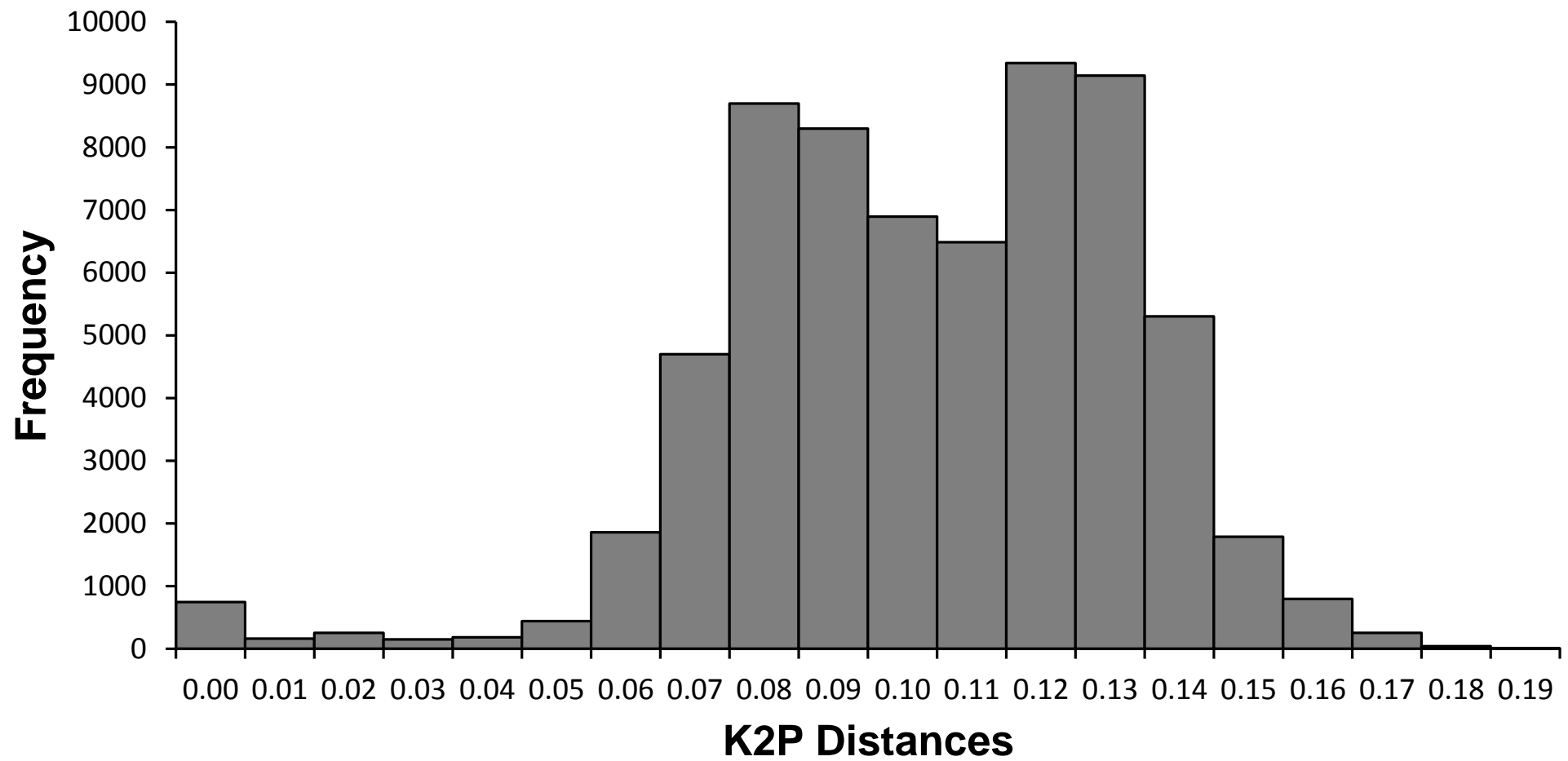

Supplement: Fig S7 — Pairwise distances (K2P) for 502 full-length (654bp) DNA barcode sequences from Australian Hypertrophinae. [file men0014-0706-SD7.pdf]

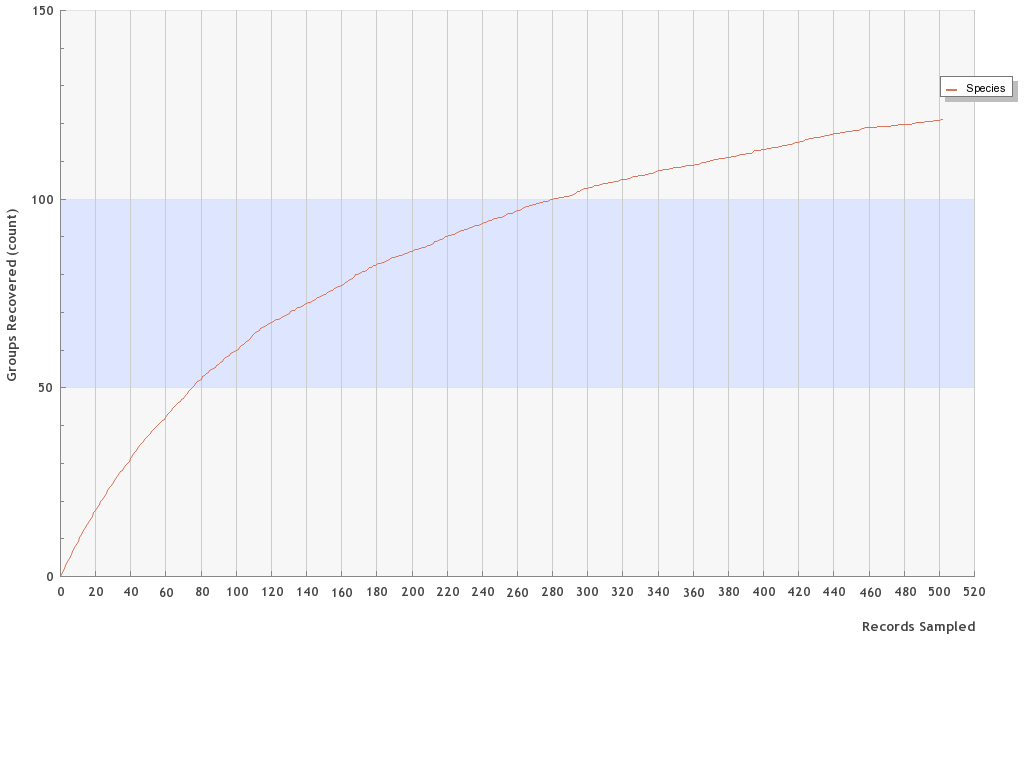

Supplement: Fig S9 — An accumulation curve of putative species (OTUs). [file men0014-0706-SD9.png]
